# Phase I study of plitidepsin in combination with bortezomib and dexamethasone in patients with relapsed/refractory multiple myeloma

**DOI:** 10.1002/cam4.5250

**Published:** 2022-09-20

**Authors:** María Victoria Mateos, Felipe Prosper, Jesús Martin Sánchez, Enrique M. Ocio, Albert Oriol, Cristina Motlló, Jean‐Marie Michot, Isidro Jarque, Rebeca Iglesias, María Solé, Sara Martínez, Carmen Kahatt, Salvador Fudio, Gema Corral, Ali Zeaiter, Lola Montilla, Vincent Ribrag

**Affiliations:** ^1^ Hospital Universitario de Salamanca Salamanca Spain; ^2^ Clínica Universidad de Navarra Pamplona Spain; ^3^ Centro de Investigación Biomédica en Red de Cáncer (CIBERONC) Instituto de Salud Carlos III Madrid Spain; ^4^ Hospital Universitario Virgen del Rocio Sevilla Spain; ^5^ Hospital Universitario Marqués de Valdecilla (IDIVAL) Universidad de Cantabria Santander Spain; ^6^ Institut Català d'Oncologia Hospital Germans Trias i Pujol Badalona Spain; ^7^ Institut Gustave Roussy Villejuif France; ^8^ Hospital Universitario La Fe Valencia Spain; ^9^ M.D. Anderson Cancer Center Madrid Spain; ^10^ PharmaMar Madrid Spain

**Keywords:** bortezomib, dexamethasone, multiple myeloma, phase I study, plitidepsin

## Abstract

Previous studies showed antitumor activity for plitidepsin plus dexamethasone (DXM) in relapsed/refractory multiple myeloma (r/r MM), and in vitro synergism with bortezomib (BTZ) or DXM against MM cells. This phase I trial evaluated plitidepsin (3‐h intravenous infusion Day 1 and 15), BTZ (subcutaneous bolus Day 1, 4, 8, and 11), and DXM (orally Day 1, 8, 15, and 22), every 4 weeks in 36 r/r MM patients. Twenty‐two patients were treated using a standard dose escalation design (10 at the recommended dose [RD] cohort), and 14 additional patients were treated to expand the RD cohort. No dose‐limiting toxicities (DLTs) occurred during dose escalation. The highest dose level evaluated (plitidepsin 5.0 mg/m^2^, BTZ 1.3 mg/m^2^, DXM 40.0 mg) was the RD for phase II studies. Results shown herein are focused on this RD. Two patients had DLTs (grade 3 diarrhea, and grade 3 nausea/vomiting refractory to antiemetic therapy). Grade ≥ 3 hematological toxicity (thrombocytopenia 46%, anemia 33%, and neutropenia 17%) was manageable and did not result in treatment discontinuation. Transient and manageable grade 3 ALT increase (26%) was the most common biochemical abnormality. At the RD cohort, overall response rate was 22.2% (95%CI, 6.4%–47.6%), including one stringent complete response, one very good partial response, and two partial responses in r/r patients to BTZ and/or lenalidomide. The clinical benefit rate was 77.8% (95%CI, 52.4–93.6%). No major pharmacokinetic drug–drug interaction was found. In conclusion, the triple combination of plitidepsin, BTZ, and DXM showed an acceptable safety profile and had moderate activity in adult patients with r/r MM.

## Graphical abstract

This phase I trial evaluated antitumor activity for plitidepsin plus bortezomib (BTZ) and dexamethasone (DXM) in relapsed/refractory multiple mieloma patients (r/r MM). This triple combination of plitidepsin, BTZ, and DXM showed an acceptable safety profile and had moderate activity in adult patients with r/r MM that have failed to standard common therapeutic options, including BTZ and lenalidomide.

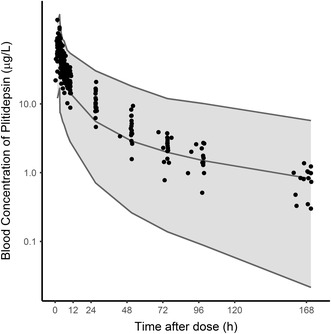

## 1 INTRODUCTION

Multiple myeloma (MM) is a B‐cell malignancy accounting for 10% of hematological malignant diseases worldwide, with about 120,000 new cases every year^1^ and a 5‐year survival rate of 57%.^2^ With current induction regimens, complete response is achieved in >60% of newly diagnosed MM patients, especially when combined with autologous stem cell transplantation.^3^ In relapsed/refractory MM (r/r MM), new drugs added to available treatment options include second‐generation immunomodulatory drugs (IMiDs) (pomalidomide), and proteasome inhibitors (carfilzomib, ixazomib). New family drugs such as monoclonal antibodies (daratumumab, isatuximab, elotuzumab), peptide‐drug conjugate alkylating agents (Melphalan flufenamide), XP01‐inhibitors (selinexor), BCMA antibody‐drug conjugate (Belantamab mafodatin) and BCMA‐targeted CAR‐T cells therapy (Abecma) have also been approved.^4^, ^5^, ^6^, ^7^ Despite these advancements, MM remains generally incurable and no standard cure is currently available for patients with r/r MM previously treated with multiple regimens including proteasome inhibitors, IMiDs, and anti‐CD38 monoclonal antibodies.

The feasibility of re‐treating MM patients with single‐agent bortezomib (BTZ) was first confirmed in the phase III VISTA trial,^8^ where 47% of patients pre‐treated with BTZ‐based regimens during earlier phases of the disease responded again to the drug alone. This was further supported by the results of the phase II RETRIEVE and SUMMIT trials and the phase III APEX trial.^9^, ^10^, ^11^ Combinations of BTZ with other drugs (e.g., IMiDs, panobinostat, or monoclonal antibodies) also induced response in MM patients after failing previous treatment with BTZ.^12^, ^13^, ^14^, ^15^, ^16^ However, despite these promising results, new regimens based on BTZ combined with drugs with different mechanisms of action that may reverse resistance to BTZ are a medical need.

Plitidepsin is a cyclic depsipeptide originally extracted from the tunicate *Aplidium albicans* and currently produced by chemical synthesis that has potent in vitro activity against primary MM tumor cells and a broad spectrum of human MM cell lines.^17^ Plitidepsin interacts with the eukaryotic elongation factor eEF1A2, an oncogene overexpressed in MM cells.^18^ This interaction leads to early oxidative stress, Rac1 activation, initiation of the MAPK cascade and cyclin down‐regulation, induction of endoplasmic reticulum stress, and activation/deactivation of several cytoplasmic factors. These events finally result in induction of apoptosis, perturbation of the cell cycle, and a potent antiproliferative effect. In the ADMYRE phase III trial, plitidepsin combined with dexamethasone (DXM) showed antitumor activity against r/r MM (overall response rate [ORR]) of 13.8% with a median duration of response of 12 months. The median progression‐free survival (PFS) was 3.8 months and safety profile was acceptable.^19^ Plitidepsin in combination with DXM was approved in Australia in December 2018 for the treatment of patients with r/r MM who have received at least three prior treatment regimens, including both a proteasome inhibitor and an IMiD. Plitidepsin plus DXM has also been approved for use after two prior lines of therapy if disease is refractory and/or intolerant to both a proteasome inhibitor and an IMiD.^20^ In this setting, further trials are needed to better define the role of plitidepsin in combination with other active agents in patients with MM.

In vitro, synergistic activity was found for the combination of plitidepsin with BTZ or DXM in MM cell lines and primary MM cells.^17^ The positive results observed with plitidepsin and BTZ against MM (both as single agents and in combination with DXM), their different mechanisms of action, and the potential synergism of the triple combination warranted the exploration of the efficacy of plitidepsin plus BTZ and DXM in a clinical trial in patients with relapsed and/or refractory MM.

The objective of this phase I clinical trial was to determine the recommended dose (RD) for phase II studies, the activity and safety profile, and the pharmacokinetics (PK) of plitidepsin in combination with BTZ and DXM in patients with r/r MM.

## 2 METHODS

This study was conducted at six centers in Spain and one in France according with the Declaration of Helsinki, Good Clinical Practice guidelines, and local regulations for clinical trials. The study protocol was approved by the Independent Local Ethics Committee of each center. Signed written informed consent was obtained for each patient prior to any procedure.

### 2.1 Patient population

Inclusion criteria were age ≥ 18 years; r/r MM diagnosed according to Durie‐Salmon criteria; ≥1 previous treatment lines; recovery from previous toxicities to grade ≤ 1 (excluding alopecia); Eastern Cooperative Oncology Group performance status score ≤ 2; normal left ventricular ejection fraction (LVEF); platelet count ≥50 × 10^9^/L; hemoglobin ≥8.0 g/dL; absolute neutrophil count ≥1.0 × 10^9^/L (lower values accepted if severe bone marrow [BM] infiltration); and adequate hepatic and renal function. Patients pretreated with BTZ or another proteasome inhibitor could be included if they had achieved a minor response (MR) >2 months at least once (patients who became BTZ refractory in subsequent therapies could be also included).

### 2.2 Study treatment

Treatment consisted of escalating doses of plitidepsin 3‐h intravenous (i.v.) infusion Day 1 and 15, and BTZ subcutaneous bolus injection Day 1, 4, 8, and 11, with fixed DXM dose 40.0 mg orally Day 1, 8, 15, and 22, all every 4 weeks (q4wk), for a maximum of 8 cycles. If response or disease stabilization was observed, treatment after Cycle 8 could continue with plitidepsin and DXM at the same doses. Antiemetic prophylaxis was given to all patients, consisting of ondansetron 8 mg i.v. or equivalent, diphenhydramine hydrochloride 25 mg i.v. or equivalent, and ranitidine 50 mg i.v. or equivalent administered 30 min before each plitidepsin infusion. If necessary, 10 mg of metoclopramide every 8 h could be administered after the end of plitidepsin infusion or the duration of treatment with serotonin antagonists could be extended.

### 2.3 Dose escalation

Dose escalation followed a standard 3 + 3 design. The starting plitidepsin dose (4.0 mg/m^2^) was 80% of the RD for plitidepsin alone as a 3‐h i.v. infusion Day 1 and 15 q4wk.^21^ The starting BTZ dose (1.0 mg/m^2^) was about 80% of the approved dose (1.3 mg/m^2^) in relapsed MM as a twice‐weekly schedule, alone or in combination with DXM.^22^

### 2.4 Study assessments

Hematology and biochemistry tests were done at baseline, before each plitidepsin or BTZ administration during Cycle 1, and before each plitidepsin administration during subsequent cycles.

Adverse events (AEs) and laboratory abnormalities were graded using the National Cancer Institute Common Terminology Criteria for Adverse Events (NCI‐CTCAE) v.4. Antitumor activity was evaluated according to the International Myeloma Working Group (IMWG) criteria.^23^ Efficacy endpoints comprised ORR (percentage of patients with stringent complete response [sCR], complete response [CR], very good partial response [VGPR] and partial response [PR]), the percentages of patients with MR and stable disease (SD), the clinical benefit rate (percentage of patients with ORR plus MR or SD), PFS, and duration of response (DoR).

### 2.5 Pharmacokinetic analyses

Sixteen samples were collected from each patient to quantify plitidepsin whole blood and BTZ plasma concentrations at baseline and at different times 1 week after the first infusion. In patients treated at the RD, 15 additional samples were taken 1 week after the second plitidepsin infusion.

### 2.6 Statistical analyses

Continuous variables were presented with summary statistics and categorical variables in frequency tables. Time‐to‐event variables were calculated using Kaplan–Meier approach. Binomial exact distribution was used to calculate 95% confidence intervals (95%CIs) for categorical variables. Individual PK parameters were tabulated. Statistical analyses were performed using SAS v.9.2, (SAS Institute Inc), and PK non‐compartmental analysis (NCA) was performed using Phoenix WinNonlin v.6.3.

## 3 RESULTS

### 3.1 Patient characteristics and treatment

A total of 39 patients were enrolled between June 2014 and August 2018. Of these, 36 patients were treated at three dose levels: eight at DL1 (plitidepsin 4.0 mg/m^2^, BTZ 1.0 mg/m^2^ and DXM 40.0 mg), four at DL2 (plitidepsin 4.0 mg/m^2^, BTZ 1.3 mg/m^2^, and DXM 40.0 mg), and 10 at DL3 (recommended dose [RD] for this combination) (plitidepsin 5.0 mg/m^2^, BTZ 1.3 mg/m^2^, and DXM 40.0 mg) plus 14 additional treated patients to expand the RD cohort. An additional three patients at the RD cohort were included but never treated because they unmet the required criteria before starting treatment on Cycle 1 Day 1. Twelve of the 22 treated patients at the dose escalation stage were evaluable for the determination of the RD (data presented at the ASCO 2016 Annual Meeting).^24^

Of the 39 enrolled patients at all dose levels, with median age 66 years (range, 51–80 years), 19 (49%) had previously received hematopoietic stem cell transplantation (HSCT) (12 patients, 44%, at the RD) (Table 1). The median number of previous chemotherapy lines was 4 (range, 1–9 lines) at all dose levels (5 lines, range, 1–9 lines, at the RD). At all dose levels, 24 of 39 patients (62%) were refractory to last prior therapy (16 patients, 59%, at the RD). At all dose levels, 20 of 39 patients (51%) were refractory to IMiDs agents. Eleven patients (28%) were refractory to BTZ at all dose levels; 10 of these 11 patients (91%) were refractory to BTZ plus IMiDs, and one (9%) only to BTZ. At the RD, 12 of 27 patients (44%) were refractory to IMiDs agents, and nine (33%) to BTZ; eight of these nine patients (89%) were refractory to BTZ plus IMiDs, and one (11%) only to BTZ (Table 1).

**TABLE 1:** Baseline characteristics of included patients

	RD cohort (*n* = 27)	Total (*n* = 39)
Gender		
Male	15 (56%)	21 (54%)
Female	12 (44%)	18 (46%)
Median age, years (range)	64 (51–80)	66 (51–80)
ECOG performance status		
0	5 (19%)	8 (21%)
≥1	22 (81%)	31 (79%)
MM type at diagnosis		
Non‐secretory/Oligosecretory	2 (7%)	3 (8%)
Secretory	25 (93%)	36 (92%)
IgG	18 (67%)	25 (64%)
IgA	4 (15%)	6 (15%)
Lambda light‐chain disease	2 (7%)	3 (8%)
Kappa light‐chain disease	1 (4%)	2 (5%)
Durie‐Salmon stage at diagnosis		
I^a^	1 (4%)	3 (8%)
II^b^	6 (22%)	12 (31%)
III^c^	20 (74%)	24 (62%)
ISS stage at diagnosis		
I	8 (30%)	14 (36%)
II	5 (19%)	6 (15%)
III	7 (26%)	10 (26%)
Disease status with respect to last prior therapy^d^		
Relapsed	11 (41%)	15 (39%)
Total refractory	16 (59%)	24 (62%)
Disease status with respect to last prior agents		
Refractory to BTZ	9 (33%)	11 (28%)
Refractory to IMiDs	12 (44%)	20 (51%)
Best response to last prior anticancer therapy		
CR	1 (4%)	2 (5%)
VGPR	2 (7%)	7 (18%)
PR	9 (33%)	12 (31%)
SD	2 (7%)	4 (10%)
PD	8 (30%)	9 (23%)
UK	5 (19%)	5 (13%)
Prior HSCT		
Autologous	10 (37%)	17 (44%)
Autologous and allogenic	2 (7%)	2 (5%)
No. of lines of prior chemotherapy		
Median (range)	5 (1–9)	4 (1–9)
1	4 (15%)	6 (15%)
2	1 (4%)	7 (18%)
3	2 (7%)	3 (8%)
4	3 (11%)	5 (13%)
5 or more	17 (63%)	18 (46%)
Prior anticancer agents		
BTZ	26 (96%)	35 (90%)
Lenalidomide	25 (93%)	34 (87%)
Thalidomide	14 (52%)	16 (41%)
Pomalidomide	16 (59%)	17 (44%)
Carfilzomib	9 (33%)	9 (23%)
Daratumumab	9 (33%)	9 (23%)
Panobinostat	1 (4%)	1 (3%)

Abbreviations: BTZ, bortezomib; CR, complete response; ECOG, Eastern Cooperative Oncology Group; HSCT, hematopoietic stem cell transplantation; Ig, immunoglobulin; IMiDs, immunomodulatory; ISS, International Staging System; MM, multiple myeloma; MR, minor response; PD, progressive disease; PR, partial response; RD, recommended dose; SD, stable disease; UK, unknown; VGPR, very good partial response.

^a^ All stage IA.

^b^ Includes stage IIA (*n* = 11; 5 at the RD) and stage IIB (*n* = 1, at the RD).

^c^ Includes stage IIIA (*n* = 16, 13 at the RD) and stage IIIB (*n* = 8, 7 at the RD).

^d^ Relapsed MM: previously treated myeloma that progressed and required salvage therapy but did not meet the criteria for either “refractory” or “relapsed and refractory” myeloma. Total refractory MM includes both, “refractory MM” and “relapsed and refractory MM”. *Refractory MM*: disease that was non‐responsive in patients who had never achieved a MR or better with any therapy. *Relapsed and refractory MM*: disease that was non‐responsive while on salvage therapy, or progressed within 60 days of the last therapy in patients who had achieved MR or better with any therapy.

A total of 227 cycles were administered at all dose levels (median of 3 cycles per patient, range, 1–29 cycles); 111 of these cycles were given at the RD cohort, with a median of 2 cycles per patient (range, 1–29 cycles). Median relative dose intensity at the RD was 73% for both plitidepsin and BTZ, and 68% for DXM.

### 3.2 Dose‐limiting toxicities and recommended dose

Twelve of the 22 patients treated at the escalation stage were evaluable for dose‐limiting toxicities (DLTs).

The other10 patients were non‐evaluable for RD. Six of these patients did not receive a complete Cycle 1 (two patients discontinued before ending Cycle 1 due to pneumonia unrelated to treatment, one due to early PD, one due to plitidepsin hypersensitivity reaction, one patient had treatment omission due to treatment‐related grade 1 nausea and vomiting, and one patient due to missed oral DXM dose). Of the other four patients, two received incorrect BTZ or DXM doses at Cycle 1, one received treatment on Days 4 and 11 despite not fulfilling treatment criteria, and one had no laboratory tests done in Cycle 1.

No DLTs occurred during dose escalation. Dose escalation beyond DL3 was not allowed by the study protocol; hence, DL3 (plitidepsin 5.0, BTZ 1.3 mg/m^2^, and DXM 40.0 mg) was defined as the RD for this combination, and then 14 additional patients were treated to expand the RD cohort.

At the RD cohort, 12 of the 24 treated patients were evaluable for DLTs. Two patients (17%) had DLTs during Cycle 1; these DLTs consisted of grade 3 diarrhea, and a combination of grade 3 nausea and grade 3 vomiting refractory to antiemetic therapy, in one patient each.

### 3.3 Toxicity profile

The most common treatment‐related AEs found among the 36 patients treated at all dose levels were nausea (36% of patients), fatigue (28%), diarrhea (25%), and peripheral neuropathy and vomiting (19% each). Most of these AEs were grade 1/2. Grade ≥ 3 treatment‐related AEs at all dose levels comprised fatigue (*n* = 3 patients; 8%), diarrhea and vomiting (*n* = 2; 6% each), and constipation, nausea, plitidepsin extravasation, hypersensitivity, and respiratory tract infection (*n* = 1 each; 3%).

Among the 24 patients treated at the RD cohort, the most frequent treatment‐related AEs were nausea (33% of patients), diarrhea (25%), fatigue (21%), and vomiting (17%). Most of these AEs were grade 1/2 (Table 2). Treatment‐related grade ≥ 3 AEs at the RD cohort consisted of diarrhea in two patients (8%), and nausea, vomiting, fatigue, respiratory tract infection and plitidepsin extravasation in one patient (4%) each.

**TABLE 2:** Treatment‐related adverse events (>10% of patients) and laboratory abnormalities at the RD cohort

	Plitidepsin 5.0 mg/m^2^, BTZ 1.3 mg/m^2^ and DXM 40.0 mg (RD cohort)							
	Per patient (*n* = 24)				Per cycle (*n* = 111)			
NCI‐CTCAE grade	1–2	3	4	Total	1–2	3	4	Total
Hematological laboratory abnormalities								
Anemia	16 (67%)	7 (29%)	1 (4%)	24 (100%)	94 (86%)	9 (8%)	1 (1%)	104 (95%)
Thrombocytopenia	9 (38%)	5 (21%)	6 (25%)	20 (83%)	50 (45%)	15 (14%)	7 (6%)	72 (66%)
Neutropenia	9 (38%)	4 (17%)	—	13 (54%)	20 (18%)	9 (8%)	—	29 (26%)
Biochemical laboratory abnormalities (per patient)								
ALT increased (*n* = 23)	15 (65%)	6 (26%)	—	21 (91%)	78 (72%)	10 (9%)	—	88 (81%)
AST increased (*n* = 23)	18 (78%)	1 (4%)	—	19 (83%)	52 (49%)	1 (1%)	—	53 (50%)
Creatine increased (*n* = 22)	14 (64%)	1 (5%)	—	15 (68%)	13 (13%)	1 (1%)	—	14 (14%)
CPK increased (*n* = 22)	7 (32%)	1 (5%)	—	8 (36%)	41 (39%)	1 (1%)	—	42 (40%)
Adverse events								
Nausea	7 (29%)	1 (4%)	—	8 (33%)	10 (9%)	1 (1%)	—	11 (10%)
Diarrhea	4 (17%)	2 (8%)	—	6 (25%)	16 (14%)	2 (2%)	—	18 (16%)
Fatigue	4 (17%)	1 (4%)	—	5 (21%)	33 (30%)	2 (2%)	—	35 (32%)
Vomiting	3 (17%)	1 (4%)	—	4 (17%)	3 (3%)	1 (1%)	—	3 (4%)
Extravasation	—	1 (4%)	—	1 (4%)	—	2 (2%)	—	2 (2%)
Respiratory tract infection	—	1 (4%)	—	1 (4%)	—	2 (2%)	—	2 (2%)

*Note*: The number of patients/cycles with each adverse event is specified. Laboratory abnormalities are shown regardless of relationship to treatment. Other treatment‐related grade 3 adverse events were extravasation and respiratory tract infection in one patient each.

Abbreviations: ALT, alanine aminotransferase; AP, alkaline phosphatase; AST, aspartate aminotransferase; BTZ, bortezomib; CPK, creatine phosphokinase; NCI‐CTCAE, National Cancer Institute Common Terminology Criteria for Adverse Events; RD, recommended dose.

The most common hematological abnormality (regardless of relationship to treatment) at all dose levels was anemia (all patients). Most cases were grade 1/2. Grade ≥ 3 anemia was found in 11 of 36 treated patients (31%). Thrombocytopenia was observed in 86% of patients reaching grade ≥ 3 in 17 patients. Neutropenia was found in 47% of patients with grade ≥ 3 in five patients. No episodes of febrile neutropenia were reported.

At the RD cohort, grade ≥ 3 anemia was present in eight of 24 treated patients (33%; grade 4 in one). Thrombocytopenia was found in 83% of patients with grade ≥ 3 in 11 patients (46%; grade 4 in six). Neutropenia was found in 54% of patients, with grade 3 in four patients (Table 2).

Most patients with grade ≥ 3 hematological abnormalities at the RD already had abnormal blood cell counts (grade ≥ 2) at baseline, including six of 11 with grade 3/4 thrombocytopenia, three of four with grade 3 neutropenia, and seven of eight with grade 3/4 anemia. No treatment discontinuations, cycle delays, or dose reductions occurred at the RD due to hematological toxicity, which was managed with dose omissions.

The most frequent biochemical abnormality regardless of relationship was alanine aminotransferase (ALT) increase (94%) at all dose levels, followed by aspartate aminotransferase (AST) increase (80%), creatine increase (71%), and creatine phosphokinase (CPK) increase (35%). Most transaminase increases at all dose levels were grade 1/2, and none reached grade 4. Grade 1/2 ALT increases and AST increases were reported in 26 patients each (46% of patients had grade 1 ALT increase and 57% had grade 1 AST increase). Grade 3 ALT increases were reported in seven patients (20%) and grade 3 AST increase occurred in two patients (6%).

Among the 24 patients treated at the RD cohort, the most frequent biochemical abnormality regardless of relationship was ALT increase (91%), followed by AST increase (83%), creatine increase (68%), and CPK increase (36%). Most of these abnormalities at the RD cohort were grade 1/2 and none reached grade 4; grade 3 were ALT increase in six patients, and grade 3 AST increase, creatinine increase and CPK increase in one patient each (Table 2).

At the RD cohort, three treatment discontinuations occurred due to non‐hematological toxicity (grade 3 diarrhea in Cycle 2, grade 3 nausea and vomiting refractory to antiemetic therapy [DLTs] in Cycle 1, and grade 3 ALT/AST increase in Cycle 2). Six patients had dose reductions in 7 cycles. Of these, five dose reductions occurred due to toxicity, with four of them involving plitidepsin (two episodes of grade 3 ALT increase, and one episode each of grade 3 fatigue, and grade 2 CPK increase) and only one involving BTZ (grade 1 paresthesia). Two dose reductions were due to reasons unrelated to treatment. Ten patients required packed red blood cell transfusions and six were given platelet transfusions.

### 3.4 Efficacy

Twenty‐eight patients treated at all dose levels (18 at the RD cohort) were evaluable for efficacy as per IMWG criteria. Eight patients were not evaluable because they did not receive at least one complete cycle. Overall, 10 patients showed PR or better, including two sCRs, one CR, three VGPRs, and four PRs (ORR = 35.7%; 95%CI, 18.6–55.9%). The clinical benefit rate was 82.1% (95%CI, 63.1%–93.9%) (Table 3).

**TABLE 3:** Antitumor activity according to IMWG criteria in relapsed/refractory multiple myeloma patients

	Plitidepsin (mg/m^2^) + BTZ (mg/m^2^) + DXM (mg) dose level						Total (*n* = 28)	
	Dose level 1 4.0/1.0/40.0 (*n* = 7)		Dose level 2 4.0/1.3/40.0 (*n* = 3)		Dose level 3 (RD cohort) 5.0/1.3/40.0 (*n* = 18)			
	*n*	%	*n*	%	*n*	%	*n*	%
sCR	—	—	1	33.3	1	5.6	2	7.1
CR	1	14.3	—	—	—	—	1	3.6
VGPR	2	28.6	—	—	1	5.6	3	10.7
PR	1	14.3	1	33.3	2	11.1	4	14.3
MR	1	14.3	1	33.3	1	5.6	3	10.7
SD ≥4 months	1	14.3	—	—	2	11.1	3	10.7
SD <4 months	—	—	—	—	7	38.9	7	25.0
PD	1	14.3	—	—	4	22.2	5	17.9
ORR^a^ (95%CI)	57.1% (18.4–90.1%)		66.7% (9.4–99.2%)		22.2% (6.4–47.6%)		35.7% (18.6–55.9%)	
Clinical benefit rate^b^ (95%CI)					77.8% (52.4–93.6%)		82.1% (63.1–93.9%)	

*Note*: Data shown are patients evaluable for efficacy at all dose levels.

Abbreviations: BTZ, bortezomib; CR, complete response; DXM, dexamethasone; IMWG, International Myeloma Working Group; MR, minor response; ORR, overall response rate; PD, progressive disease; PR, partial response; RD, recommended dose; sCR, stringent complete response; SD, stable disease; VGPR, very good partial response.

^a^ ORR includes sCR, CR, VGPR, and PR.

^b^ Clinical benefit rate includes sCR, CR, VGPR, PR, MR, or SD.

At the RD, one sCR, one VGPR, and two PRs occurred (ORR = 22.2%; 95%CI, 6.4%–47.6%). Both PRs were observed in refractory patients (one patient was refractory to lenalidomide and the other one to BTZ, lenalidomide, and pomalidomide). The clinical benefit rate was 77.8% (95%CI, 52.4–93.6%) (Table 3).

At all dose levels, the median DoR was 14.4 months (95%CI, 1.8 months‐23.9 months) and the median PFS was 4.1 months (95%CI, 2.1–10.4 months). At the RD cohort, the median DoR was 12.3 months (95%CI, 1.8–23.9 months) and the median PFS was 2.8 months (95%CI, 1.2–4.8 months).

### 3.5 Pharmacokinetics

All patients were sampled for PK analysis, but only 17 and 18 of them were suitable for NCA of plitidepsin and BTZ, respectively. Mean total body clearance (CL) and volume of distribution (*V*_ss_) for plitidepsin at DL1, DL2, and DL3 were 5.67, 5.59, and 9.41 L/h and 169, 192, and 341 L, respectively (Table 4). Regarding BTZ, the mean values at DL1, DL2, and DL3 for maximum plasma concentration (C_max_) were 11.0, 16.9, and 17.0 μg/L, respectively; for total clearance after extravascular administration (CL/F) they were 23.4, 17.9, and 24.9 L/h; and for volume of distribution at terminal phase (V_z_/F) they were 1308, 1208, and 1658 L. The reference for BTZ PK results was a phase I study in patients with MM.^25^

**TABLE 4:** Non‐compartmental pharmacokinetic parameters

	Plitidepsin (mg/m^2^) + BTZ (mg/m^2^) + DXM (mg) dose level			BTZ (mg/m^2^)
	Dose level 1 4.0/1.0/40.0	Dose level 2 4.0/1.3/40.0	Dose level 3 (RD cohort) 5.0/1.3/40.0	1.3
Plitidepsin	*n* = 5	*n* = 3	*n* = 9	—
*C*_max_ (μg/L)	83.8 (26.3)	117 (55.1)	71.7 (18.4)	—
AUC (h* μg/L)	1263 (393)	1420 (538)	1064 (332)	—
HL (h)	40.1 (20.0)	46.2 (10.4)	44.6 (16.2)	—
CL (L/h)	5.67 (1.52)	5.59 (2.54)	9.41 (4.64)	—
*V*_ss_ (L)	169 (68.6)	192 (88.0)	341 (208)	—
*V*_*z*_ (L)	185 (56.3)	394 (254)	671 (620)	—

BTZ	*n* = 6	*n* = 3	*n* = 9	*n* = 10
*C*_max_ (μg/L)	11.0 (7.71)	16.9 (4.21)	17.0 (8.87)	16.5 (8.4)
AUC (h*μg/L)	77.7 (17.9)	134 (46.8)	138 (88.6)	151 (53.5)
HL (h)	39.1 (9.22)	51.5 (41.4)	49.0 (17.8)	65.7 (46.5)
CL/*F* (L/h)	23.4 (6.79)	17.9 (6.03)	24.9 (19.2)	16.6 (5.8)
*V*_*z*_/*F* (L)	1308 (431)	1208 (52.4)	1658 (1457)	1330 (578)

*Note*: Values are expressed as mean (standard deviation). Plitidepsin and BTZ by dose level on Day 1 of Cycle 1 in the present study and of BTZ in a reference study (see Moreau et al^25^).

Abbreviations: AUC, area under the concentration–time curve from time zero to infinity; BTZ, bortezomib; CL, total clearance; CL/F, total clearance of drug after extravascular administration, corrected for absolute bioavailability; *C*_*max*_, maximum concentration; DXM, dexamethasone; HL, terminal half‐life; MM, multiple myeloma; RD, recommended dose; *V*_ss_, volume of distribution at steady‐state; *V*_*z*_, apparent volume of distribution during terminal phase; *V*_*z*_/*F*, apparent volume of distribution during terminal phase after extravascular administration, corrected for absolute bioavailability.

## 4 DISCUSSION

The RD for phase II studies for this triple combination was plitidepsin 5.0 mg/m^2^ on Day 1 and 15, BTZ 1.3 mg/m^2^ on Day 1, 4, 8, and 11, and DXM 40.0 mg on Day 1, 8, 15, and 22 q4wk. This triple combination was generally well tolerated, with no DLTs observed during dose escalation. At the expanded cohort, two patients had DLTs: one with grade 3 diarrhea, and one with grade 3 nausea/vomiting refractory to antiemetic therapy.

The most common hematological abnormality (all grades) was anemia, which occurred in all patients (grade ≥ 3 in 33% at the RD). Most cases were grade 1/2, and at the RD the majority of patients with grade ≥ 3 anemia while on treatment already had grade 2 anemia at baseline. The most common severe hematological toxicity at the RD was thrombocytopenia, which was grade ≥ 3 in half of patients with most of them (6 of 11) already having grade ≥ 2 thrombocytopenia at baseline. Lower rates of severe thrombocytopenia (21%–22%) have been previously observed in patients with r/r MM treated with the double combination of plitidepsin 5.0 mg/m^2^ on Day 1 and 15 q4wk plus DXM in phase II and III trials.^19^, ^26^ Transient thrombocytopenia is common in r/r MM patients treated with single‐agent BTZ^9^, ^22^, ^27^ or with BTZ‐based combination therapies.^13^, ^14^, ^28^ Therefore, the increased incidence of thrombocytopenia found here with this triple combination compared to plitidepsin plus DXM may be attributed to the addition of BTZ. Nevertheless, grade 3 hematological abnormalities were overall transient and manageable with dose omissions and packed red blood cells or platelet transfusions; none of them required treatment discontinuation.

The most common biochemical abnormality was transaminase increases. Grade 3 ALT increase was most frequent at the RD (26% of patients), but it was transient and manageable with dose delays, omissions, or reductions.

Treatment‐related AEs at the RD for this triple combination were mostly mild/moderate; grade 3 diarrhea, nausea, vomiting, or fatigue were observed in less than 10% of patients. Severe muscular toxicity was not observed with the triple combination in this study. In contrast, incidences of 5.4% and 3.6% were reported for severe myalgia and muscular weakness, respectively, in MM patients treated with plitidepsin plus DXM.^19^ Furthermore, some severe toxicities associated with BTZ‐containing combinations, such as peripheral neuropathy,^28^ or with IMiDs, such as respiratory infections only present in one patient,^29^, ^30^, ^31^, ^32^ were absent or with a low rate at the RD for this triple combination. We cannot discard that these toxicities could occur in a larger patient population or among patients treated for longer periods.

At the RD cohort, only three patients discontinued treatment due to treatment‐related events, thereby suggesting an acceptable safety profile for plitidepsin combined with BTZ and DXM in this heavily pretreated r/r MM population (pretreated with a median of five prior lines, HSCT in 44% and refractory disease in 59%).

This triple combination showed modest antitumor activity (ORR of 35.7% at all dose levels; 22.2% at the RD) in this population of heavily pretreated r/r MM patients, most of them having received BTZ and with disease refractory to the last prior therapy. Comparing the efficacy of the 18 evaluable patients during the escalation stage with an ORR of 55.6% (data presented at the ASCO 2016 Annual Meeting),^24^ a drop to 35.7% is now observed after the expansion stage. Some differences were observed in the characteristics of patients treated at the expanded RD cohort compared to those patients treated in the other two dose levels. Patients at the RD were more heavily pretreated, with a median of five prior lines per patient versus two prior lines per patient at DL1 and DL2. At the RD, 33% of patients were pretreated with daratumumab or carfilzomib and 59% with pomalidomide, whereas no patients were treated with these drugs at DL1 or DL2, except for one patient treated with pomalidomide at DL2. Furthermore, 59% of patients at the RD were refractory to last line (one patient was refractory only to BTZ, six to IMiDs agents, and eight to both agents).

The antitumor activity found in patients treated with the combination of plitidepsin, BTZ, and DXM (ORR of 35.7% at all dose levels, and 22.2% at the RD) was higher than that observed in the ADMYRE trial evaluating plitidepsin plus DXM (ORR: 13.8%),^19^ but lower than that reported in studies with other BTZ‐based regimens, such as the phase III VISTA trial,^8^ where 47% of patients (pre‐treated with BTZ‐based regimens during earlier phases of the disease) responded again to the drug alone.

Results of the VISTA trial were further supported by those of phase II trials: RETRIEVE (ORR: 40%) and SUMMIT (ORR: 35%, including minimal response while on BTZ treatment), and of the phase III APEX trial (crossover from DXM to BTZ), in which the overall and complete response rates with BTZ were 43% and 9%, respectively.^9^, ^10^, ^11^

Of note, the median DoR (14.4 months at all dose levels; 12.3 months at the RD) was longer than that observed with most other BTZ and DXM‐containing combinations, including panobinostat (6.0 months),^33^ pomalidomide (7.4 months),^13^ lenalidomide (8.7 months),^12^ venetoclax (9.7 months),^34^ or elotuzumab (11.4 months).^15^ Compared to plitidepsin, BTZ, and DXM, the triple combinations based on BTZ and DXM with lenalidomide or elotuzumab are associated with higher incidences of grade 3/4 non‐hematological adverse events, such as peripheral neuropathy, pulmonary events, and infections.^12^, ^15^

**FIGURE 1:**
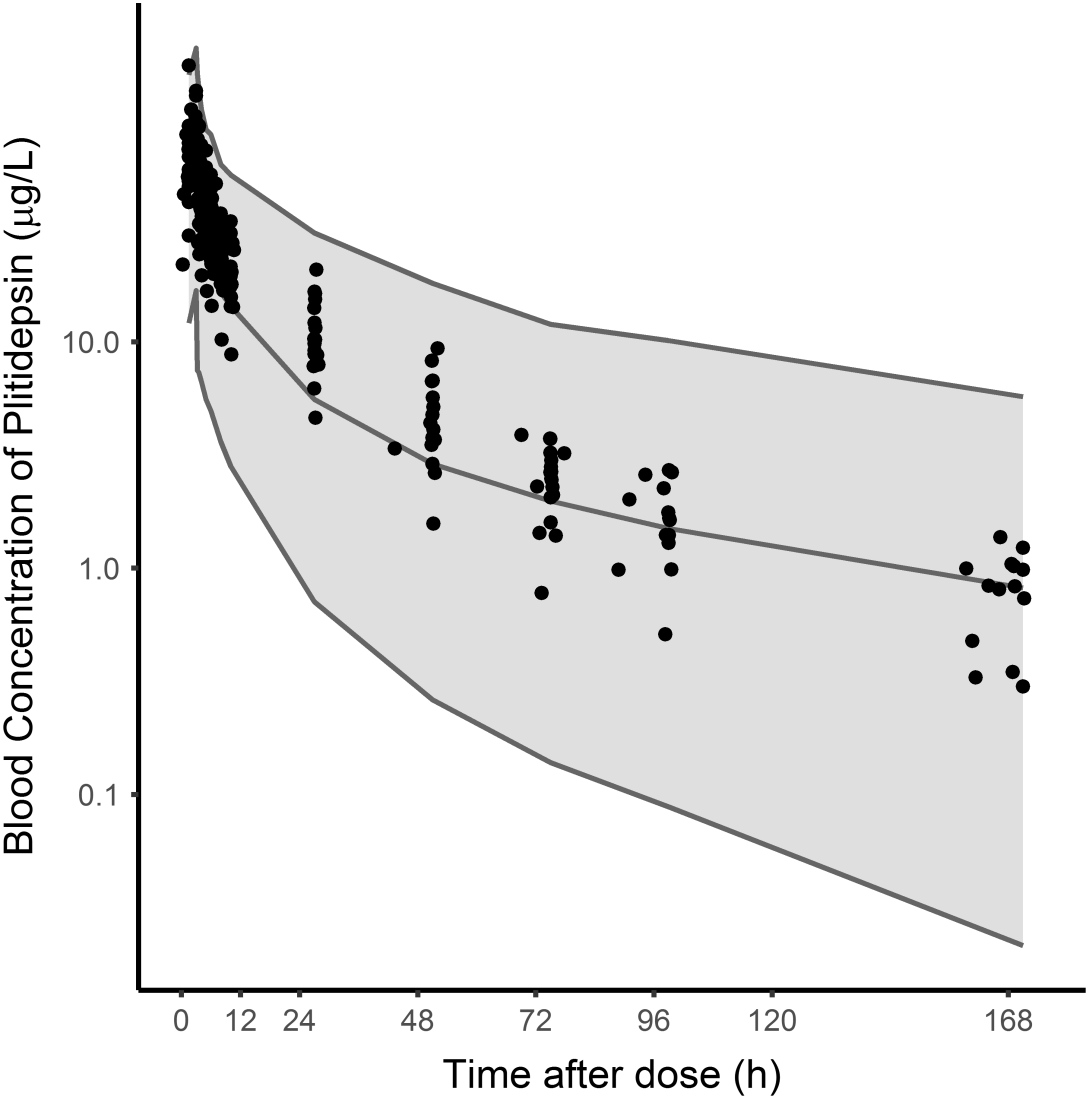
Typical plitidepsin exposure in patients with in relapsed/refractory multiple myeloma patients and observed plitidepsin concentrations. Dots represent observations from the current study, lines represent the 5th, 50th, and 95th percentile of the simulated concentrations, and the shaded area represents the 90% prediction interval of plitidepsin whole blood concentrations

The similarity of PK parameters found in the present study compared to reference values, and the lack of overlapping metabolic routes of plitidepsin and BTZ, suggest that no major PK interaction between these two drugs is expected. Compared to the values reported herein, patients with MM treated with plitidepsin in other trials had a similar median CL (4.4 L/h) and a somewhat larger median peripheral volume (513 L) (unpublished data). The plitidepsin concentrations found in this study fitted within the 90% prediction interval simulated for patients with MM, although most were above the 50th percentile for the typical values expected during the first 48 hours after dosing (Figure 1). Regarding BTZ, values reported herein were comparable to those reported elsewhere (16.5 μg/L, 16.6 L/h, and 1330 L, respectively) for a dose of 1.3 mg/m^2^ administered subcutaneously and with the same sampling schedule duration of 72 hours.^25^

In summary, the triple combination of plitidepsin 5.0 mg/m^2^ on Day 1 and 15, BTZ 1.3 mg/m^2^ on Day 1, 4, 8, and 11, and DXM 40.0 mg on Day 1, 8, 15, and 22 q4wk was generally well tolerated and had moderate activity (ORR of 22.2%) in patients with r/r MM that have failed to standard common therapeutic options, including BTZ and lenalidomide.

## AUTHOR CONTRIBUTIONS

María Victoria Mateos: Conceptualization, Investigation, Resources, Writing—original draft, Writing—review & editing. Felipe Prosper: Investigation, Resources, Writing—review & editing. Jesús Martin Sánchez: Investigation, Resources, Writing—review & editing. Enrique M. Ocio: Methodology, Investigation, Resources, Writing—review & editing. Albert Oriol: Investigation, Resources, Writing—review & editing. Cristina Motlló: Investigation, Resources, Writing—review & editing. Jean‐Marie Michot: Investigation, Resources, Writing—review & editing. Isidro Jarque: Investigation, Resources, Writing—review & editing. Rebeca Iglesias: Investigation, Resources, Writing—review & editing. María Solé: Investigation, Resources, Writing—review & editing. Sara Martínez: Conceptualization, Methodology, Writing—original draft, Writing—review & editing, Supervision. Carmen Kahatt: Conceptualization, Methodology, Writing—review & editing, Supervision. Salvador Fudio: Conceptualization, Methodology, Formal analysis, Writing—original draft, Writing—review & editing, Supervision. Gema Corral: Methodology, Formal analysis, Writing—review & editing. Ali Zeaiter: Methodology, Writing—review & editing, Supervision. Lola Montilla: Methodology, Writing—original draft, Writing—review & editing. Vincent Ribrag: Investigation, Resources, Writing—review & editing.

## FUNDING INFORMATION

The study was funded by Pharma Mar, S.A.

## CONFLICT OF INTEREST

María Victoria Mateos has received honoraria for lectures and participation in advisory boards from Janssen, Celgene, Takeda, Amgen, GSK, AbbVie, Pharma Mar, Pfizer, Regeneron, Roche, Sanofi, Oncopeptides, and Seagen. Enrique M. Ocio has received honoraria or consultation fees from Janssen, BMS/Celgene, Sanofi, GSK, Oncopeptides, Takeda, Amgen, Pfizer, Karyopharm, and Pharma Mar. Jean‐Marie Michot has declared Principal/sub‐Investigator role of clinical trials from AbbVie, Agios, Amgen, Argen‐x, Astex, AstraZeneca, Beigene, Blueprint, BMS, Boeringer Ingelheim, Celgene, Chugai, Clovis, Daiichi Sankyo, Debiopharm, Eisai, Eos, Exelixis, Forma, Gamamabs, Genentech, Gortec, GSK, H3 biomedecine, Incyte, Innate Pharma, Janssen, Kura Oncology, Kyowa, Lilly, Loxo, Lysarc, Lytix Biopharma, Medimmune, Menarini, Merus, MSD, Nanobiotix, Nektar Therapeutics, Novartis, Octimet, Oncoethix, Oncopeptides AB, Orion, Pfizer, Pharmamar, Pierre Fabre, Roche, Sanofi, Seattle Genetics, Servier, Sierra Oncology, Taiho, Takeda, Tesaro, and Xencor. He has also received personal fees such as honoraria, royalties or fees for consulting, lectures, speakers bureau, expert testimony, employment, advisory boards, among others from Roche, AstraZeneca, and Amgen. In addition, he has also received non‐financial support such as, equipment supplied by the entity, travel paid by the entity, writing support, administrative support, among other from Celgene, Bristol‐Myers Squibb, and GSK. Isidro Jarque has declared speakers bureau and consultancy role by AbbVie, Amgen, AstraZeneca, Bristol‐Myers Squibb, Gilead, Janssen, Novartis, Pfizer, Roche and Takeda. Carmen Kahatt, Salvador Fudio, Gema Corral and Ali Zeaiter report personal fees for salary as full time employee and stock ownership from Pharma Mar, outside the submitted work. Sara Martínez and Lola Montilla report personal fees for salary as full time employee from Pharma Mar, outside the submitted work. No disclosures were reported by the other authors.

## Supporting information


Appendix S1

## Data Availability

Individual participant data are not publicly available since this requirement was not anticipated in the study protocol considering that this trial started patient enrolment in 2014. Clinical trial summary results were placed in the European Clinical Trials Database (EudraCT; study 2013–003835‐31) and Clinical Trials.gov (Identifier: NCT02100657).
