# Supplementary material for: Phase I study of plitidepsin in combination with bortezomib and dexamethasone in patients with relapsed/refractory multiple myeloma
Source: Cancer Med. 2022 Sep 20;12(4):3999–4009. doi: 10.1002/cam4.5250 (PMC9972151; doi:10.1002/cam4.5250)
Supplement: Supplementary file 1 — Appendix S1 [file CAM4-12-3999-s001.docx]

**SUPPORTING INFORMATION**

**Supplementary methods**

**Exclusion Criteria**

Patients were excluded if they had been pretreated with plitidepsin; had any of the following: active/metastatic primary malignancy other than MM, plasma cell leukemia, any prior serious systemic disorders or relevant cardiac conditions, any clinical situation causing significant and persistent creatine phosphokinase (CPK) increase, disease-related symptomatic hypercalcemia despite optimal therapy, and sequelae of any prior neuropathy; were pregnant or lactating women; were receiving concomitant medications potentially active against MM; or had human immunodeficiency or hepatitis virus infection; and hypersensitivity to BTZ, polyoxyl 35 castor oil or mannitol.

**Dose-limiting Toxicities**

Dose-limiting toxicities (DLTs) were defined as grade ≥3 neutropenia (grade 4 if severe BM infiltration) with fever or lasting >7 days, grade ≥3 thrombocytopenia (grade 4 if severe BM infiltration) with grade ≥3 hemorrhage, grade ≥3 nausea/vomiting refractory to antiemetic therapy, grade ≥3 muscular toxicity, grade ≥3 transaminase increase >1 week, grade ≥3 bilirubin increase, grade ≥3 CPK increase, symptomatic or treatment-requiring plitidepsin-related grade ≥1 cardiac arrhythmia, plitidepsin-related grade ≥1 left ventricular systolic dysfunction, BTZ-related neuropathic pain and peripheral sensory neuropathy (if it resulted in BTZ discontinuation), and any grade ≥3 toxicity.

**Pharmacokinetic Analyses**

Both drugs (plitidepsin and BTZ) were measured by validated liquid extraction methods followed by ultra-performance liquid chromatography tandem mass-spectrometry detection (Dynakin, Derio, Spain). The calibration range for plitidepsin and BTZ was 0.25-100 ng/mL, and 0.1-5 ng/mL, respectively. The reference for plitidepsin PK results was a population-PK model of plitidepsin (data from 303 patients treated in seven phase II studies and one phase III study), which was used to simulate exposure in whole blood after one 3-hour i.v. infusion of 5.0 mg/m² of plitidepsin in patients with similar characteristics compared to those treated in this study (unpublished data). The reference for BTZ PK results was a phase I study in patients with MM (see Moreau et al ([25](#_ENREF_25))).
